# Senescence and carryover effects of reproductive performance influence migration, condition, and breeding propensity in a small shorebird

**DOI:** 10.1002/ece3.3533

**Published:** 2017-11-15

**Authors:** Chelsea Weithman, Daniel Gibson, Kelsi Hunt, Meryl Friedrich, James Fraser, Sarah Karpanty, Daniel Catlin

**Affiliations:** ^1^ Department of Fish and Wildlife Conservation Virginia Tech Blacksburg VA USA

**Keywords:** breeding propensity, carryover effects, Missouri River, piping plover, senescence

## Abstract

Breeding propensity, the probability that an animal will attempt to breed each year, is perhaps the least understood demographic process influencing annual fecundity. Breeding propensity is ecologically complex, as associations among a variety of intrinsic and extrinsic factors may interact to affect an animal's breeding decisions. Individuals that opt not to breed can be more difficult to detect than breeders, which can (1) lead to difficulty in estimation of breeding propensity, and (2) bias other demographic parameters. We studied the effects of sex, age, and population reproductive success on the survival and breeding propensity of a migratory shorebird, the piping plover (*Charadrius melodus*), nesting on the Missouri River. We used a robust design Barker model to estimate true survival and breeding propensity and found survival decreased as birds aged and did so more quickly for males than females. Monthly survival during the breeding season was lower than during migration or the nonbreeding season. Males were less likely to skip breeding (range: 1–17%) than females (range: 3–26%; β_sex_ = −0.21, 95% CI: −0.38 to −0.21), and both sexes were less likely to return to the breeding grounds following a year of high reproductive success. Birds that returned in a year following relatively high population‐wide reproductive output were in poorer condition than following a year with lower reproductive output. Younger adult birds and females were more likely to migrate from the breeding area earlier than older birds and males; however, all birds stayed on the breeding grounds longer when nest survival was low, presumably because of renesting attempts. Piping plovers used a variety of environmental and demographic cues to inform their reproduction, employing strategies that could maximize fitness on average. Our results support the “disposable soma” theory of aging and follow with predictions from life history theory, exhibiting the intimate connections among the core ecological concepts of senescence, carryover effects, and life history.

## 1 INTRODUCTION

Life history theory suggests that individual iteroparous animals should balance the energetic costs of reproduction and the rearing of young with future survival and long‐term fitness (McNamara & Houston, 1996). Survival and reproduction require similar resources, such that the most adaptive strategy may involve trade‐offs among these functions (Stearns, 1989; Williams, 1966). The underlying assumption is that reproduction has non‐negligible costs (Harshman & Zera, 2007), and thus, to not breed is to conserve energy for survival and future breeding (Reznick, Bryant, & Bashey, 2002; Ricklefs & Wikelski, 2002; Robinson et al., 2010). Trade‐offs among demographic rates associated with fitness may be exacerbated by “carryover effects”, which suggests that previous conditions can affect the states of individuals into the future (Harrison, Blount, Inger, Norris, & Bearhop, 2011; Norris, 2005; Norris & Marra, 2007; O'Connor, Norris, Crossin, & Cooke, 2014; Sedinger, Schamber, Ward, Nicolai, & Conant, 2011). Such carryover effects have been observed to impact individual reproductive effort and success in a suite of species with a range of life history strategies (Lachish, McCallum, & Jones, 2009; Reid, 1987; Warren et al., 2014).

Breeding propensity, or the probability that an animal will attempt to breed each year, is perhaps the least understood demographic process influencing annual fecundity (Etterson et al., 2011). Fecundity often is expressed as the number of young produced per female of reproductive age in the population (Etterson et al., 2011), yet variation in behavior may leave some breeding females undetected (Olson et al., 2005). Individuals that choose not to breed in a given year also may be more difficult or impossible to detect, as they may not be physically or behaviorally available for detection (Sedinger, Lindberg, & Chelgren, 2001). As organisms age, their reproductive costs may increase (Proaktor, Milner‐Gulland, & Coulson, 2007), and their survival prospects may decrease (Nussey, Froy, Lemaitre, Gaillard, & Austad, 2013), and thus the optimal decision to breed or not also should change through time. In addition to these intrinsic changes in potential fitness, studies of breeding propensity have found that extrinsic factors such as food availability, population density, and predation threat also have an effect on breeding propensity (Blomberg, Gibson, Atamian, & Sedinger, 2017; Hoy, Millon, Petty, Whitfield, & Lambin, 2016; Reed, Gauthier, & Giroux, 2004; Sedinger et al., 2001).

In many taxa, reproductive strategies differ between the sexes, where the costs of display, ornamentation, and territory setup and defense often fall primarily to males (Clutton‐Brock & Isvaran, 2007), while females bear the largest share of the direct costs of reproduction (Nager, Monaghan, & Houston, 2001). Other reproductive activities such as incubation (Lengyel, Kiss, & Tracy, 2009), feeding, vigilance, and defense (Reznick et al., 2002; Robinson et al., 2010; Walters, 1984) are variously shared between the sexes, presumably as an evolutionarily stable strategy between the sexes. Indeed, the effort expended by male animals in polygynous societies often leads to reduced lifespans when compared to females (Clutton‐Brock & Isvaran, 2007; Nussey et al., 2013). Each individual faces a variety of decisions and trade‐offs in all demographic processes, including breeding propensity, that balance short‐term gains with long‐term fitness prospects (Nicolai & Sedinger, 2012).

**Figure 1:**
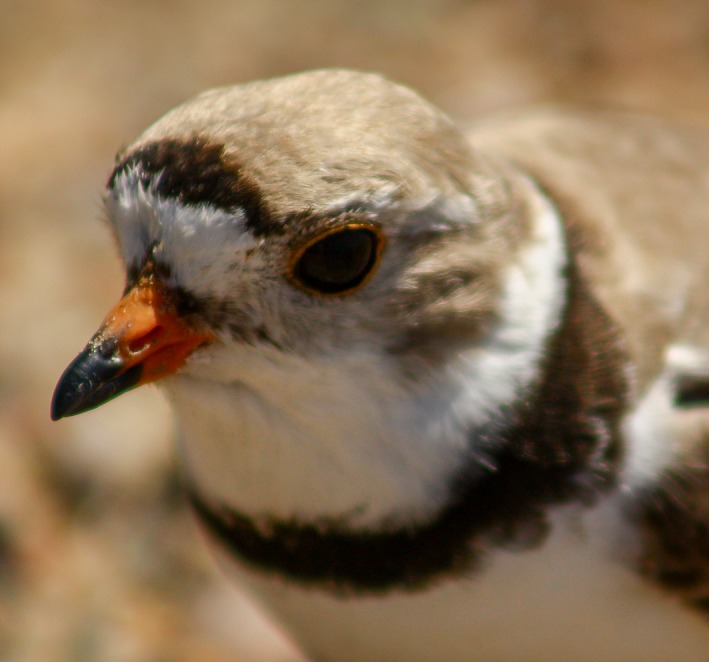
Female piping plover on the Missouri River. Photograph by Diane Borden

In this study, we used resights of individually marked male and female piping plovers (*Charadrius melodus*; hereafter “plovers”; Figure 1) from a breeding population along the Missouri River and throughout their nonbreeding range along the southern Atlantic and Gulf coasts of North America to study the link between life history and breeding decisions. Plovers are conspicuous, temperate breeding, ground‐nesting shorebirds that exhibit high levels of fidelity to breeding and wintering sites (Catlin, Fraser, & Felio, 2015; Friedrich, Hunt, Catlin, & Fraser, 2015), making them ideal subjects for the study of carryover effects and breeding propensity. Plover chicks are precocial, but adults brood and monitor their young after hatch (Elliot‐Smith & Haig, 2004), which leaves adults subject to predation and trading their own maintenance for vigilance (Walters, 1984). Male and female plovers have different levels of investment throughout the reproductive cycle. Male plovers establish and conspicuously defend territories where females lay clutches in small depressions in the sand. The sexes share incubation and early brooding and defense, but the females often leave before the young fledge, leaving the male to monitor the young until fledging (Elliot‐Smith & Haig, 2004). Some females will attempt to breed a second time if successful, typically with a new mate, but the frequency of these matings is exceedingly low in most years (Hunt et al., 2015).

Changes in habitat, individual condition, and population density have been shown to reverberate across seasons in a variety of taxa, by affecting or precluding reproduction, leading to delayed departures and arrivals from nonbreeding locations, and a host of other effects (Harrison et al., 2011; Norris & Marra, 2007). Plovers are territorial throughout the annual cycle, competing for and protecting nesting sites and both breeding and nonbreeding season feeding territories, a behavior that could put late‐arriving breeders at a disadvantage. A variety of studies have shown that conditions during the nonbreeding season can affect arrival times on the breeding grounds as well as reproductive success (Harrison et al., 2011; Norris & Marra, 2007). Piping plovers that arrive to the breeding grounds and nest earlier, on average, have offspring with higher growth and survival rates, and, as with many birds, their offspring may also have higher fitness (Blums, Clark, & Mednis, 2002; Catlin et al., 2015; Saunders, Roche, Arnold, & Cuthbert, 2012; Verhulst & Nilsson, 2008), suggesting there is a significant fitness cost for birds that breed later in the season.

Overall, we hypothesized that birds would act to maximize their long‐term fitness (Williams, 1966), while optimizing short‐term gains where possible (Nicolai & Sedinger, 2012). We predicted that classes of birds with higher average mortality rates would be less likely to skip breeding than those with lower mortality rates. As senescence is fairly common, albeit difficult to detect (Nussey et al., 2013), we predicted that plover survival would decrease as birds aged, and accordingly, breeding propensity would increase with age. Because the bulk of territorial defense and chick rearing falls to males, we hypothesized that female plovers would have higher survival leading to generally lower breeding propensity than male plovers (Clutton‐Brock & Isvaran, 2007).

In addition to the life history linkages, we predicted that the physiological cost of successfully rearing a brood to fledging would negatively impact breeding propensity (McNamara & Houston, 1996). Moreover, we hypothesized that the resultant increased competition for resources with a larger hatch‐year cohort would carry over and affect the subsequent year's population‐wide reproductive success. We hypothesized that increased effort by plovers in 1 year would manifest in later average arrival at the nonbreeding locations because of the significant investment of time and energy involved in successfully rearing a brood. Therefore, we also predicted that the average departure time from the nonbreeding locations would be later after years with relatively high reproductive output. Finally, we hypothesized that these multiple effects would be detectable in the average adult condition (in this case, measured as body mass), such that following a year with relatively high reproductive output, the average adult condition would be lower.

## 2 MATERIALS AND METHODS

### 2.1 Study area and field methods

**Figure 2:**
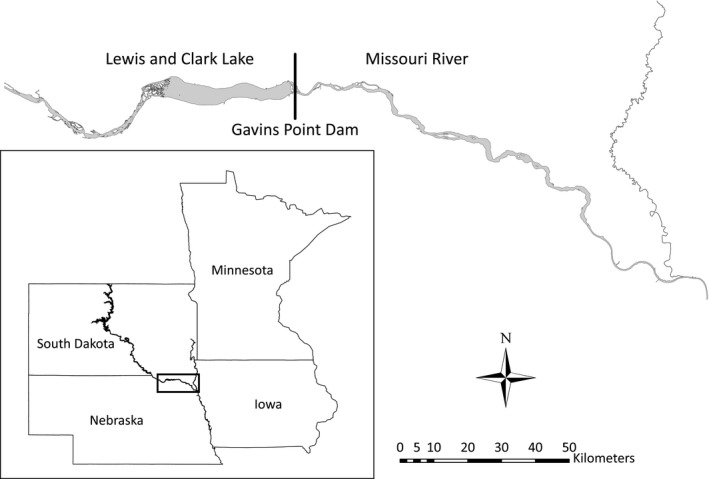
Map of the study area on the Missouri River in South Dakota and Nebraska, USA

We collected breeding season (April–August, 2005–2014) data on the Missouri River near the Gavins Point Dam (42°51′N, 97°29′W) and Lewis and Clark Lake (42°51′N, 97°47′W; Figure 2). We located nests on sandbars and checked them approximately every other day until hatch or failure. Breeding adult birds and recently hatched young were marked during each year of the study, and incubating (approximately 2–26 days after clutch completion) adults were recaptured and weighed each year where possible. Previous analyses have determined that there was negligible variation in masses relative to time since clutch completion (K. Hunt, unpublished data); therefore, we used these masses as an index to the condition of breeding birds (Labocha & Hayes, 2012). Only those chicks that survived and returned to the study area were used in this analysis. We attempted to resight all marked birds every 2 days throughout the breeding season (approx. April 10–Aug 15). Migration and nonbreeding season resights (“auxiliary resights”; August–April, 2005–2014) of marked birds were both collected by us and contributed by others from the southern Atlantic and Gulf coasts of North America (Foster, Amos, & Fuiman, 2009; Gibson et al., 2017; Gratto‐Trevor et al., 2012; Roche et al., 2010). For detailed information on the study area and field methods, see Ref. Catlin et al. (2015).

### 2.2 Analytical methods

#### 2.2.1 Arrival and departure times on wintering grounds

To explore the functional connection between breeding success and the following year's breeding propensity, we modeled the effect of population‐level, average reproductive output on wintering arrival, and departure dates (first and last reported sightings, respectively, on the wintering grounds), using the same individuals used in the breeding propensity analysis. We used mixed linear regression to assess the effect of reproductive output and minimum‐known age (each bird was assigned age = 1 the first time it was seen or captured as an adult, even if it was initially captured as a chick in another year, hereafter, “age”; 1–10 year) on arrival and departure times on the wintering grounds. Less than 35% of the birds in our sample of adults were initially captured as chicks (i.e., known age); thus, we assigned them minimum‐known age for parity with the remainder of the sample. A random intercept effect for individual was included to control for multiple measures of individual birds. We compared a fully time variable (year) model, to one with time‐varying covariates replacing year and a null model. Because reproductive output is a year‐specific variable and therefore redundant with a “year” effect, we could not include it and the year variable in the same model. We reasoned that with enough data, the time‐varying model would be the best‐fitting model (as reproductive output is only one factor potentially contributing to annual variation). Thus, we used the analysis of deviance test (Skalski, 1996) to determine the proportion of variation in arrival and departure times that is described by reproductive output.

#### 2.2.2 Condition

In addition to arrival and departure times, we examined the relationship between reproductive success and adult condition (mass) the following year. We used a mixed, linear regression to assess the effect of reproductive output and age (minimum known) on adult condition. A random intercept effect for individual was included to control for multiple measures of individual birds. We used the same method as above to determine the proportion of variation in condition described by reproductive output (Skalski, 1996).

#### 2.2.3 Survival modeling

To estimate survival and breeding propensity, we used the robust design Barker model proposed by Kendall et al. (2013). This model allowed us to estimate survival and both temporary and permanent emigration. Robust design models use secondary sampling occasions within longer, primary occasions to refine estimates of recapture, allowing for the estimation of temporary emigration, thus separating nondetections from absences (Pollock, 1982). The Barker (1997) model makes use of auxiliary resightings and recoveries to estimate fidelity and a relatively unbiased (i.e., less affected by emigration) estimate of survival. The robust design Barker model includes nine estimable parameters and one derived parameter (N, Table 1). We used a Huggins closed‐capture formulation (Huggins, 1991) of the robust design model to estimate N, so this parameter was derived and not part of the likelihood function.

**Table 1:** Descriptions of the parameters in the robust design Barker model used in this study

Parameter	Description
*S*	Probability an individual survives from one primary occasion to the next
*F*	Probability an individual remains in the study population between primary occasions, given that it survives that occasion
*a′*	Probability of returning from an unobservable state in a primary occasion given that an individual was unavailable for capture in the previous primary occasion (i.e., reimmigration)
*a″*	Probability of remaining available for recapture given that an individual was available for capture in the previous primary occasion (i.e., inverse of temporary emigration)
*p*	Probability that an individual is first detected in a secondary occasion given that it is alive and available for capture
*c*	Probability that an individual is captured within a primary occasion given that it is alive, available for capture, and was captured in a previous secondary occasion within that primary occasion
*r*	Probability that an individual is reported dead between primary occasions (there were no dead recoveries in our study, so this parameter was fixed to 0 for all analyses)
*R*	Probability that an individual is detected alive between primary periods and survives to the following primary period
*R′*	Probability that an individual is detected alive between primary periods but does not survive to the following primary period
*N*	Population size during a primary period (derived from other parameters in the model)

#### 2.2.4 Temporal structure

**Figure 3:**
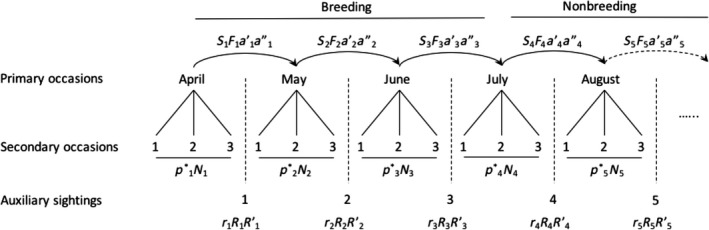
Structure of the Barker robust design model as implemented for this study. The model comprises five 30‐day primary sampling occasions, beginning 15 April and ending 12 September, and an approximately 215‐day period lasting until the following 15 April. Each of the 30‐day primary occasions consists of three 10‐day secondary occasions, and each primary occasion is accompanied by an auxiliary period. The model is broken into two overall periods, breeding and nonbreeding. The parameters associated with each primary (*S*—survival rate, *F*—fidelity rate, *a′*—temporary immigration, *a″*—temporary emigration, and *N*—the derived population size), secondary (p*—the combined capture (*p*) and recapture rate (*c*) for secondary periods), and auxiliary (*r*—recovery rate, *R* and *R′*—resight rates offsite) periods are shown

Robust design capture–mark–recapture studies consist of shorter, secondary occasions, between which the population is assumed to be closed (i.e., no deaths, births, immigrations, or emigrations), within longer primary occasions, between which the population is assumed to be open. We divided each breeding season (2005–2014) into five, 30‐day primary sampling occasions, beginning 15 April and ending 12 September, followed by an approximately 215‐day period lasting until the following 15 April. Each of the 30‐day primary occasions consisted of three 10‐day secondary occasions (Figure 3). Based on relatively high estimates of site fidelity in this population (Catlin et al., 2015; Friedrich et al., 2015) and timing of migration (D. Catlin, personal observation), we assumed that process by which birds became available for capture or not roughly described the migration and arrival process. As such, we forced all individuals to enter the unobservable state (migration and nonbreeding) between the 4th and 5th primary occasions (July to August) each year by fixing both *a″* (probability of remaining available, or within season fidelity) and *a′* (returning from the unobservable state) to zero. We set *p* to 1 for the final occasion within each year, indicating perfect detection. Most years, there were few or no resights on the breeding grounds during the final period because many or most adult birds had already left the population.

#### 2.2.5 Breeding propensity

We used the estimates of *a′* to develop an estimate of breeding propensity. By setting availability to 0 during the nonbreeding season (*a”* and *a′*), we estimated breeding propensity as $1-\prod_{\mathrm{April}}^{\mathrm{July}}a^{\prime }$, or 1 minus the probability of returning to the available state (breeding population) during the breeding season. To interpret this value as breeding propensity, we assumed that (1) birds that were “available for capture” would breed and (2) those that are temporarily absent for 1 year do not breed in another area. Because there were no marked birds prior to April 2005, breeding propensity estimates are only available from 2006 to 2014. Previous work indicated that few adult birds (<2.5%) emigrated to other breeding populations each year, and fewer of those returned to our study area to breed in subsequent years (Catlin et al., 2016). We do not, however, have information regarding assumption 1; thus, our estimate of breeding propensity may be higher than the realized breeding propensity. There is a possibility that this definition is sex‐biased in this system. The conspicuous displays by males attempting to breed may make them more detectable than females, regardless of their success in gaining a mate. We modeled for sex‐specific resighting rates to control for this potential bias.

#### 2.2.6 Model and variable selection

We assessed goodness‐of‐fit by decomposing our robust design Barker model into a standard Barker model, because there is no analog test for open, robust design models. We used a median $\hat{c}$ test to estimate overdispersion in our live‐recapture and recovery model with all variables estimated as time‐dependent except *r*, which we set constant at 0 because there were no dead recoveries.

To simplify modeling of the nine parameters in the robust design Barker model, we performed five stages of investigation to reduce overall computation time and reduce the number of models under consideration (Appendix S1). In brief, for the first stage, we tested multiple functional forms for several variables (*S*,* F*,* p*,* c*,* R*, and *R′*) to provide a baseline model with which we could test hypotheses related to the primary factors of interest (*S*,* a″*, and *a′*). We used an additive model (month + year + sex; where month refers to the 30‐day intervals, beginning April 15 each year) for both *a″* and *a′* at this stage and moving forward to improve estimability, and because we were interested in describing the remaining variation with time‐specific variables. We standardized the sex variable for known sex individuals (untransformed data: 1 = female, 0 = male) such that the resulting standardized mean value was 0 and the SD was 1. We then assigned individuals with unknown sex the mean value (0), which allowed us to include all individuals in our analysis without affecting the estimates associated with sex. In the second stage, we included the effect of age (minimum known) and reproductive output from the previous year on survival (*S*). In the third stage, we compared model structures for *a″* (probability of remaining available, or within season fidelity) that included variables for average nest failure during the 30‐day interval (standardized), age, and a linear trend over month. In the fourth stage, we compared model structures for *a′* (returning from the unobservable state) that contained variables for the population‐level, average reproductive output in the previous year, age, and a linear trend over month. In the fifth and final stage, we compared the model from stage 4 to models with full‐time (monthly and yearly) variability in *a″* and *a′* (month × year + sex) using the analysis of deviance (Skalski, 1996). We compared the fully time variable model to the baseline model (month + year + sex) and the baseline model with added time‐specific covariates to determine the proportion of temporal variation described by the covariate model. We used Akaike's Information Criterion corrected for small sample bias (AIC_c_) to rank and compare models in the first four stages of model development. We used the best‐fitting (lowest AIC_c_) model to estimate the specific effects of covariates (βs). Real estimates were derived from model‐averaging over all models in stage 4 (Burnham & Anderson, 2002).

## 3 RESULTS

From 2005 to 2014, we monitored 1,302 adult piping plovers: 508 males, 456 females, and 338 birds of unknown sex. Of these birds, 453 were banded as chicks and returned to the study area as adults. During the study, we monitored an average of 244 nests and 275 chicks each year (Table 2). The monthly average proportion of nests that failed from April to July was 0.23, and average reproductive output was 1.22 fledged chicks per pair per year, although both values varied considerably annually (Table 2, Catlin et al., 2015; Hunt, 2016). We did not detect any lack of fit ($\hat{c}$ = 1.0, 95% CI: 0.97–1.1) of the general Barker model.

**Table 2:** Reproductive data for piping plovers nesting on the Missouri River (2005–2014). These variables were used to describe plover survival, residency during the breeding season, and breeding propensity as covariates in the survival analysis

Year	Nests monitored	Chicks banded	Proportion of nests failing^a^	Ro^b^
2005	205	187	0.16	1.58
2006	211	218	0.23	0.84
2007	216	296	0.19	0.66
2008	295	450	0.23	1.22
2009	305	523	0.19	1.14
2010	254	100	0.30	0.83
2011	241	68	0.42	0.46
2012	178	224	0.21	1.54
2013	214	269	0.23	2.12
2014	319	418	0.09	1.78

a Average of monthly average proportions of failed nests (April–July). These values are apparent nest success.

b Reproductive output, measured as the population average of fledged chicks produced per pair (Catlin et al., 2015, K. Hunt, D. Catlin, J. Fraser unpublished data).

### 3.1 Arrival, departure, and condition

**Figure 4:**
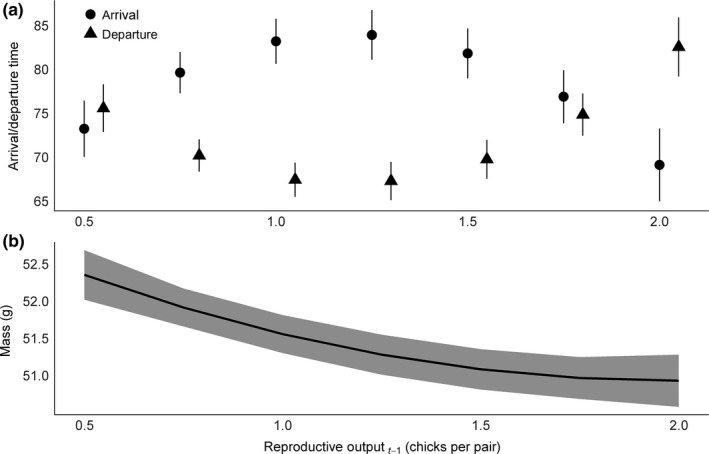
Relationship between (a) arrival to and departure from nonbreeding locations and (b) piping plover adult condition (measured as mass in g from captures and recaptures of nesting adults) with the population average reproductive output (chicks fledged per pair) from the preceding breeding season for migratory piping plovers from the Missouri River. Error bars represent 1 *SE*

The timing of arrival on the wintering grounds, departure from the wintering grounds, and the condition of individuals varied significantly over time (Table 3). Models that replaced year with variables for annual reproductive output (Ro) and reproductive output squared (Ro^2^) explained 25%, 24%, and 32% of the temporal variation in arrival, departure, and condition, respectively (Table 3). Plovers arrived at wintering sites earlier when reproductive output was relatively low or when it was relatively high (Table 4, Figure 4). Similarly, plovers departed wintering locations earlier following moderate reproductive success compared to when success was relatively low or relatively high (Figure 4). Adult condition was negatively correlated with average reproductive output in the previous breeding season, but the negative effect was less apparent at relatively high values of reproductive output (Figure 4). Females arrived earlier to wintering locations than males, but they did not depart those locations earlier, nor was their condition different from males (Table 4). The age of a bird appeared to have no effect on arrival and departure times, but condition did improve as birds aged, although the effect lessened with increasing age (age and age^2^, respectively; Table 4).

**Table 3:** Model comparisons for timing of piping plover arrival on nonbreeding locations, departure from wintering grounds, and for adult condition (mass) relative to age, sex, and population‐wide reproductive output

Factor	Model^a^	Deviance	Parameters	Variation described^b^
Arrival	Null	4600.3	5	
	Time	4513.2	15	
	Covariate	4578.1	7	0.25
Departure	Null	3099.4	5	
	Time	2990.1	14	
	Covariate	3073.0	7	0.24
Condition	Null	11367.5	5	
	Time	11219.8	15	
	Covariate	11320.2	7	0.32

a For each factor, we compared three models to determine the proportion of temporal variation that was described by reproductive output: a null model—age + age^2^ + sex, a fully time variable model—year + age + age^2^ + sex, and a model where the reproductive output variable replaced year—Ro + Ro^2^ + age + age^2^ + sex. Age—minimum‐known age of an individual in years, sex—female vs. male, and Ro—population average reproductive output (chicks fledged per pair).

b The proportion of temporal variation described by a time‐dependent covariate. Defined as follow: (Deviance_null_ − Deviance_covariate_/(Deviance_null_ − Deviance_Time_) (Skalski, 1996).

**Table 4:** Regression beta estimates for the effects of reproductive output (Ro; population average from the previous breeding season), sex, and age (minimum‐known age in years) on the timing of arrival on and departure from migratory or wintering sites, and on adult condition (mass). We used generalized, mixed regression, controlling for repeated measurements of individuals throughout the study to estimate these effects

Variable	Model					
	Arrival		Departure		Condition	
	Estimate	*SE*	Estimate	*SE*	Estimate	*SE*
Intercept	52.71	10.03	99.15	9.34	52.36	0.45
Ro	53.90	16.16	−47.72	13.49	−2.55	0.67
Ro^2^	−22.66	6.44	20.95	5.31	0.64	0.26
Sex^a^	−4.10	1.97	1.17	1.58	0.00	0.09
Age	−0.10	2.93	−2.39	3.23	0.61	0.15
Age^2^	0.01	0.37	0.25	0.43	−0.05	0.02

a Standardized for known sex individuals (1 = female, 0 = male) such that the resulting mean value was 0 and the SD was 1. We then assigned individuals with unknown sex a 0, which allowed us to include all individuals in our analysis without affecting the estimates associated with sex.

### 3.2 Survival

**Figure 5:**
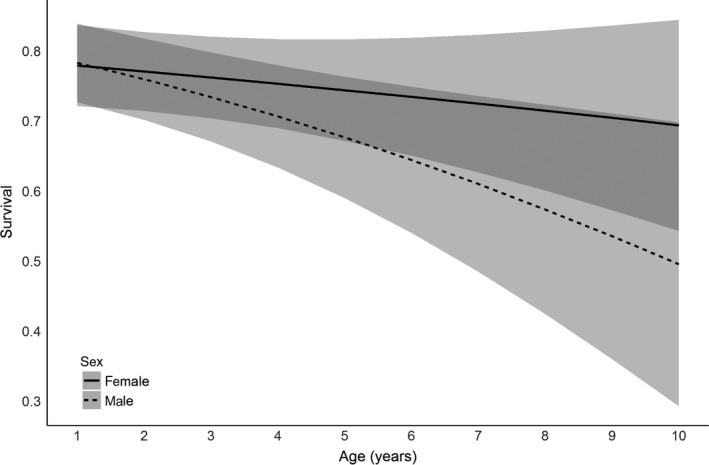
Relationship between true, annual survival and minimum‐known age for female (solid line) and male (dashed line) piping plovers on the Missouri River. Confidence bands represent 95% confidence intervals

Monthly survival varied by season and year, but was generally lower during the breeding season (${\overset{¯}{S}}_{b}$ = 0.97) than the nonbreeding season (${\overset{¯}{S}}_{n}$ = 0.98), but not significantly so in many years (Appendix S2, Figure S1). As plovers aged, monthly survival decreased (β_age_ = −0.09, 95% CI: −0.14 to −0.03) but did so more slowly for female plovers (β_age × sex_ = 0.04, 95% CI: −0.01 to 0.09; Figure 5), but there was no evidence of a difference in mean monthly survival between male and female plovers throughout the study (β_sex_ = −0.05, 95% CI: −0.22 to 0.12). Average reproductive output from the previous year did not appear to affect survival directly (β_Ro_ = −0.13, 95% CI: −0.72 to 0.46), and models containing the variable received little weight (Appendix S1, stage 2). Annual survival ranged from 0.60 to 0.84 was lowest from 2009 to 2012 and may have been lower for females than males in the final 3 years of the study (Appendix S2, Figure S2).

### 3.3 Residency

**Figure 6:**
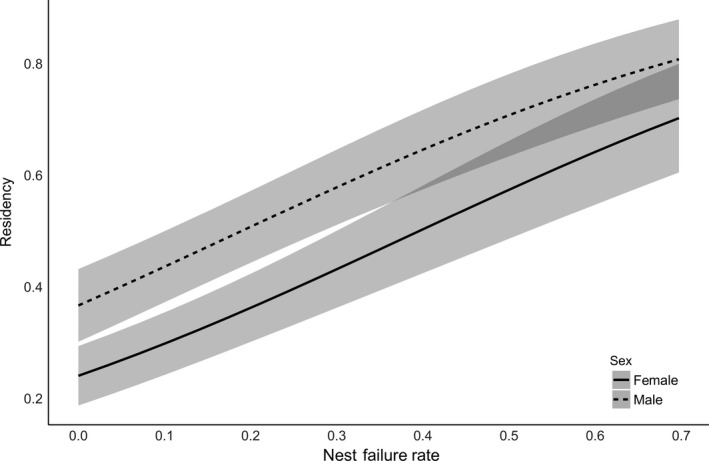
Relationship between residency (probability of remaining on site during the breeding season) and the overall population nest failure rate for female (solid line) and male (dashed line) piping plovers on the Missouri River. Confidence bands represent 95% confidence intervals, and darker regions show overlap between confidence regions between females and males

The probability that a bird remained on the breeding grounds varied by year and month, showing a downward trend as the breeding season progressed (β_Month_ = −2.22, 95% CI: −2.39 to −2.06). Female residency within a breeding season was lower than that of males (β_sex_ = −0.30, 95% CI: −0.38 to −0.21), and higher for all birds when the population nest failure rate was higher (β_nest fail_ = 0.49, 95% CI: 0.37 to 0.62; Figure 6). There was no evidence, however, that the age of a bird affected its residency time (β_age_ = 0.003, 95% CI: −0.05 to 0.06; Appendix S1, stage 3). The model containing monthly nest failure and a linear trend over the breeding season described 12% of the variation described by the fully time‐dependent model (year × month; Appendix S1, stage 5).

### 3.4 Return rate and breeding propensity

**Figure 7:**
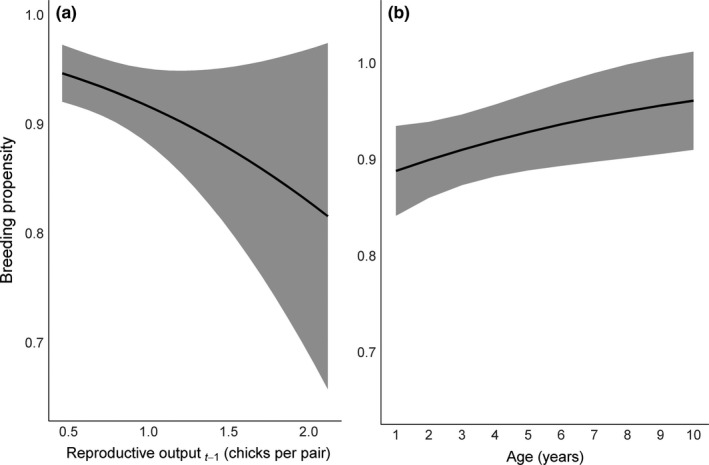
Relationship between breeding propensity (represented by the probability that an individual returns during a breeding season) and (a) the population average reproductive output (chicks fledged per pair) from the previous year and (b) minimum‐known age for piping plovers on the Missouri River. Estimates for the population mean between females and males. Confidence band represents the 95% confidence interval

**Figure 8:**
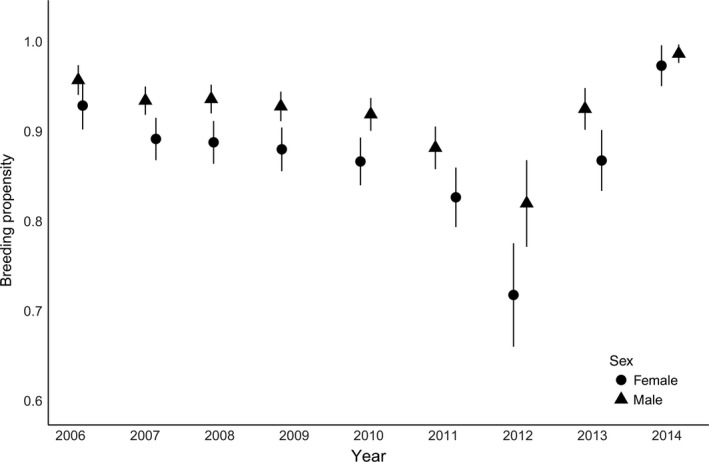
Annual breeding propensity (represented by the probability that an individual returns, or is available for detection, during a breeding season) for female (circles) and male (triangles) piping plovers on the Missouri River (2006–2014). Error bars represent 1 *SE*

Monthly return rates (and thus breeding propensity) of adults varied by year and month and were lower on average for female plovers than for males (β_sex_ = −0.21, 95% CI: −0.38 to −0.21). In addition, monthly return rates were lower following years with higher average reproductive output (β_Ro_ = −0.56, 95% CI: −1.11 to −0.21; Figure 7), but monthly return rates increased as birds aged (β_age_ = 0.10, 95% CI: 0.01–0.20; Figure 7). Breeding propensity (return rate over the entire breeding season) ranged from 0.77 to 0.99 for female plovers, and from 0.86 to 1.00 for male plovers (Figure 8). The model containing annual average reproductive output and bird age described 56% of the variation described by the fully time‐dependent model (year × month + sex; Appendix S1, stage 5).

## 4 DISCUSSION

Our results highlight the intimate connection among key ecological and life history concepts: senescence, condition, reproductive output, carryover effects, breeding propensity, and ultimately fitness. The strategies adopted by plovers in this study appeared to balance survival and breeding propensity according to predictions from life history theory in a way that would maximize fitness on average. As plovers aged, their average survival and residual fitness decreased, particularly for male plovers, and their investment in breeding condition increased. Female plovers gain fitness with increasing experience that accrues relatively early in life and males apparently do not (Saunders et al., 2012), which may explain the pattern of senescence that we observed. With age and presumably experience, female plovers' residual fitness may be balanced, whereas male plovers do not benefit from this experience and thus their residual fitness decreases more rapidly as they age.

As predicted, male plovers and older plovers of both sexes had the highest breeding propensities. The associations among a bird's condition, reproductive expenditure, and breeding propensity in this study lend support for the “disposable soma” theory of aging (Kirkwood, 1977) and suggest that individuals that were in lower quality states may have forgone breeding that year (McNamara & Houston, 1996). In fact, birds that presumably skipped breeding following a breeding attempt in the previous year, but were known alive in subsequent years (i.e., yearly capture history “101”), appeared to have had lower condition than those that were observed breeding consistently (i.e., “111”), but the sample sizes were too small for a meaningful test of that hypothesis (D. Catlin, unpublished data). If true, then the class of individuals that forego breeding in any year may represent individuals of diminished condition and reproductive state (McNamara & Houston, 1996) that are most likely to breed in years of high reproductive success, or a “bandwagon effect.”

Carryover effects of condition likely are ubiquitous; elk (*Cervus elaphus*), dark‐bellied Brent geese (*Branta bernicla bernicla*), green turtles (*Chenlonia mydas*), and plaice (*Pleuronectes platessa*) are just some of the species with evidence that condition in a previous season can affect an individual's condition and breeding in a subsequent season (Ebbinge & Spaans, 1995; Broderick, Godley, & Hays, 2001; Cook et al., 2004; Kennedy, Witthames, Nash, & Fox, 2008; reviewed in Harrison et al., 2011). The exact mechanism behind the relationship between breeding propensity and reproductive output in this study is unknown, but the arrival and departure times, as well as condition the following breeding season, indicated that there were temporal and physiological effects carried over throughout the annual cycle (Norris, 2005; Norris & Marra, 2007). Behavioral dominance can play an important role in the manifestation of carryover effects (Harrison et al., 2011). For example, dominant (typically adult male) American redstarts (*Setophaga ruticilla*) retain higher quality wintering habitats than subordinate (typically females and young individuals) birds (Norris, Marra, Kyser, Sherry, & Ratcliffe, 2004), which led to differences in condition and arrival times on their breeding grounds (Marra, Hobson, & Holmes, 1998). Plovers are territorial throughout the year, protecting nest sites in the breeding season (Elliot‐Smith & Haig, 2004) and feeding areas during the nonbreeding season (D. Catlin, personal observation), but there is no evidence of sex‐related dominance at these sites, as in redstarts (Norris et al., 2004). Therefore, if their territorial selection follows an ideal despotic distribution (Fretwell, 1972), there would be a premium on arriving early to acquire the highest quality territories at winter and breeding locations.

Seasonal interactions that lead to delayed arrival and reduced reproductive effort and success are somewhat common in birds (Norris & Marra, 2007) as well as other taxa (Harrison et al., 2011). The timing of arrival to the breeding grounds, laying date, and clutch size of pied flycatchers (*Ficedula hypoleuca*) is related to weather on wintering and staging sites (Ahola et al., 2004; Both, Bijlsma, & Visser, 2005; Laaksonen, Ahola, Eeva, Vaisanen, & Lehikoinen, 2006). For plovers, there is a clear survival and condition advantage to early breeding and thus presumably early departure from wintering location (Catlin, Milenkaya, Hunt, Friedrich, & Fraser, 2014; Catlin et al., 2015), but our study suggests that early arrival at wintering locations also has benefits. Evidence that the conditions during the breeding season can affect wintering birds is less common (Harrison et al., 2011; Norris et al., 2004), likely because it is less studied (Marra, Cohen, Loss, Rutter, & Tonra, 2015). Sedinger et al. (2011), however, showed that breeding success in black brant (*Branta bernicla nigricans*) was positively associated with occupying the highest quality winter territories, which was itself positively associated with breeding propensity the following year. These results, coupled with our study, suggest that carryover effects can be pervasive, affecting multiple seasons.

It is possible that the patterns we saw in breeding propensity, condition, and migration timing were related to population density rather than individual carryover effects (Blomberg et al., 2017; Gill et al., 2001; Stokke, Moller, Saether, Rheinwald, & Gutscher, 2005). Seasonal compensation effects act through changes in population size that affect subsequent periods through density dependence. Though not carryover effects, these compensation effects are seasonal interactions and may interact with individual carryover effects in complex ways (Harrison et al., 2011). Density is an important determinant of reproductive output for plovers in our population (Catlin et al., 2014; Hunt, 2016; Hunt et al., 2015), and it may affect other factors in their life cycle. These seasonal compensation effects, however, are positively correlated with the level of migratory connectivity or geographic linkage among populations (Norris & Marra, 2007). Plovers exhibit relatively high levels of site fidelity to both breeding and wintering locations (Friedrich et al., 2015; Gratto‐Trevor et al., 2016), but breeding populations show little connectivity with wintering populations (Gratto‐Trevor et al., 2012). Thus, the effects that we saw on wintering plovers from the previous season's reproductive output were unlikely to be related to density, but breeding propensity in subsequent years may have been related to density. The plover population we studied was positively correlated with reproductive output, increasing in size following years of relatively high success (Catlin et al., 2015; Hunt, 2016). Black‐tailed godwits (*Limosa limosa*) expanded into lower quality habitat both during the winter and during the breeding season when population sizes increased, which led to lower per capita reproduction, or a “buffer effect” (Gill et al., 2001; Gunnarsson, Gill, Petersen, Appleton, & Sutherland, 2005). If plovers reacted to increased population size similarly, higher densities in high‐quality habitat could have forced plovers into low‐quality habitat where birds were less detectable or where they would skip breeding entirely, which could explain the relationship between breeding propensity and the previous year's reproductive output.

One of the difficulties associated with studying breeding propensity is the failure of studies to detect or otherwise account for individuals that are not engaged in conspicuous breeding displays or that are not tied to a territory or breeding location (Etterson et al., 2011). Our results established for the first time for piping plovers that survival during the breeding season was on average lower than survival during the nonbreeding season, lending further support to the non‐negligible dangers inherent in breeding. In fact, this reduction in detectability offers a mechanism for the other survival differences that we observed (e.g., breeding females have lower detectability than males, nonbreeding individuals are “unavailable” for detection.). If conspicuous displays and territorial defense on the breeding grounds are costly, then we would predict that males would have lower survival and thus higher breeding propensity, which was the case in this study. Females, however, did have lower annual survival than males during the final 2 years of the study (Appendix S2, Figure S2). Although our methods are less affected by temporary emigration, even robust design models can suffer from bias in terminal estimates with substantial temporary emigration (Penaloza, Kendall, & Langtimm, 2014).

Detecting senescence in wild animals has proven difficult, which led to confusion about its prevalence in wild populations (Jones et al., 2008; Nussey et al., 2013). However, the frequency of studies that have shown either reproductive or survival senescence has increased exponentially with time (Nussey et al., 2013), and clear connections between reproductive effort and the rate of senescence have been made (Boonekamp, Salomons, Bouwhuis, Dijkstra, & Verhulst, 2014). The male‐biased pattern of senescence that we detected in this study matched predictions from life history theory. Male bias in senescence is common across multiple taxa, including humans, and appears to be positively related to the degree of polygyny found in the species (Clutton‐Brock & Isvaran, 2007; Nussey et al., 2013). Although plovers are serially monogamous, the population we studied has remarkably low mate fidelity rates (Friedrich et al., 2015), indicating that males regularly compete for mates throughout their lifetimes, subjecting them to potentially greater risk.

Our findings underscore the complex interactions between animal demography and life history, particularly for migratory, territorial species that must continually re‐establish territories within a year and across a lifetime. Decisions made in one season can have profound effects on subsequent seasons as well as lifetime fitness (Harrison et al., 2011). The cascading effects of reproductive effort carried through multiple seasons, interacted with intrinsic factors such as age and sex, and ultimately affected individual and population parameters. Our study is the first that we know of to link multiple carryover effects, including arrival and departure times, body condition, and breeding propensity through the annual cycle to describe variation in breeding performance, but such data are difficult to collect for many species. As monitoring and analytical procedures mature, ecologists will be able to understand ever more complex interseasonal interactions, allowing them to test theoretical predictions about life history trade‐offs, refining our understanding of life history and demography.

## CONFLICT OF INTEREST

None declared.

## AUTHOR CONTRIBUTIONS

CEW, DG, and DHC conceived of the ideas and designed the statistical methodology; KLH, MJF, and DHC collected the data; CEW, DHC, and DG analyzed the data; CEW and DHC led the writing of the manuscript; JDF, SMK, and DHC revised the manuscript critically for intellectual content. All authors contributed critically to the drafts and gave final approval for publication.

## DATA ACCESSIBILITY

Data for these analyses can be found at figshare.com with the following https://doi.org/10.6084/m9.figshare.4891712.
