# Supplementary material for: Senescence and carryover effects of reproductive performance influence migration, condition, and breeding propensity in a small shorebird
Source: Ecol Evol. 2017 Nov 15;7(24):11044–56. doi: 10.1002/ece3.3533 (PMC5743479; doi:10.1002/ece3.3533)
Supplement: Supplementary file 1 [file ECE3-7-11044-s001.docx]

Supplemental Information. Weithman et al. Senescence and Carryover Effects of Reproductive Performance Influence Migration, Condition, and Breeding Propensity in a Small Shorebird

**Appendix S1: Model selection and results**

The robust design Barker model estimates the following parameters: *S* – the probability an individual survives from one primary occasion to the next, *F* – the probability an individual remains in the study population between primary occasions, given that it survives that occasion, *a”* – the probability of remaining available for recapture given that an individual was available for capture in the previous primary occasion (i.e., inverse of temporary emigration), *a’* – the probability of returning from an unobservable state in a primary occasion given that an individual was unavailable for capture in the previous primary occasion (i.e., re-immigration), *p* – the probability that an individual is first detected in a secondary occasion given that it is alive and available for capture, *c* – the probability that an individual is captured within a primary occasion given that it is alive, available for capture, and was captured in a previous secondary occasion within that primary occasion, *r* – the probability that an individual is reported dead between primary occasions (there were no dead recoveries in our study, so this parameter was fixed to 0 for all analyses), *R* – the probability that an individual is detected alive between primary periods and survives to the following primary period, *R’* – the probability that an individual is detected alive between primary periods but does not survive to the following primary period, and *N* – the population size during a primary period. We used a (Huggins 1991) closed-capture formulation of the robust design model so this parameter was derived and not part of the likelihood function.

Huggins, R.M. (1991) Some practical aspects of a conditional likelihood approach to capture experiments. *BIOMETRICS,* **47,** 725–732.

To simplify modeling of the 9 parameters in the robust design Barker model, we performed 5 stages of investigation to reduce overall computation time and reduce the number of models under consideration.

*Stage 1:* For the first stage, we tested multiple functional forms for several variables (*S*, *F*, *p*, *c*, *R*, *R’*) to provide a baseline model with which we could test hypotheses related to the primary factors of interest (*S*, *a”*, *a’*).

| S | R | R' | F | p ^a^ | K ^b^ | AIC_c_ ^c^ | ΔAIC_c_ | Weight | Deviance |
| --- | --- | --- | --- | --- | --- | --- | --- | --- | --- |
| season × year + sex ^e^ | season + year | . | year | year + sex | 80 | 110755.47 | 0.00 ^f^ | 0.39 | 110594.58 |
| season × year + sex | season + year | . | . | year + sex | 71 | 110756.16 | 0.69 | 0.28 | 110613.46 |
| season × year + sex | season + year | . | year | year + sex + c | 81 | 110756.53 | 1.06 | 0.23 | 110593.62 |
| season + year + sex | season + year | . | year | year + sex | 72 | 110759.31 | 3.84 | 0.06 | 110614.59 |
| season + year + sex | season + year | . | year | year + sex + c | 73 | 110760.24 | 4.77 | 0.04 | 110613.50 |
| season + year + sex | season + year | year | year | year + sex | 80 | 110764.15 | 8.68 | 0.01 | 110603.26 |
| season + year + sex | season + year | year | year | year + sex + c | 81 | 110765.03 | 9.56 | 0.00 | 110602.12 |
| season + year + sex | season + year | . | . | year + sex | 63 | 110766.71 | 11.24 | 0.00 | 110640.16 |
| season × year + sex | season + year | . | . | year + sex + c | 72 | 110767.25 | 11.78 | 0.00 | 110622.53 |
| season + year + sex | season + year | . | . | year + sex + c | 64 | 110767.61 | 12.14 | 0.00 | 110639.04 |
| season × year + sex | season + year | year | year | year + sex + c | 89 | 110768.87 | 13.40 | 0.00 | 110589.77 |
| season + year + sex | season + year | year | . | year + sex | 71 | 110771.27 | 15.80 | 0.00 | 110628.57 |
| season + year + sex | season + year | year | . | year + sex + c | 72 | 110772.12 | 16.65 | 0.00 | 110627.40 |
| season × year + sex | season + year | year | . | year + sex | 81 | 110781.09 | 25.62 | 0.00 | 110618.18 |
| season × year + sex | season | year | year | year + sex + c | 81 | 110819.85 | 64.38 | 0.00 | 110656.94 |
| season × year + sex | season + year | year | . | year + sex + c | 80 | 110822.50 | 67.03 | 0.00 | 110661.61 |
| season + year + sex | season | year | year | year + sex | 72 | 110827.50 | 72.03 | 0.00 | 110682.78 |
| season + year + sex | season | year | year | year + sex + c | 73 | 110828.34 | 72.87 | 0.00 | 110681.60 |
| season + year + sex | season | year | . | year + sex | 63 | 110834.65 | 79.18 | 0.00 | 110708.10 |
| season + year + sex | season | year | . | year + sex + c | 64 | 110835.46 | 79.99 | 0.00 | 110706.89 |
| season × year + sex | season | year | year | year + sex | 80 | 110843.61 | 88.14 | 0.00 | 110682.72 |
| season × year + sex | season | . | year | year + sex | 71 | 110848.19 | 92.72 | 0.00 | 110705.49 |
| season × year + sex | season | . | year | year + sex + c | 72 | 110849.25 | 93.78 | 0.00 | 110704.53 |
| season + year + sex | season | . | year | year + sex | 63 | 110852.83 | 97.36 | 0.00 | 110726.28 |
| season + year + sex | season | . | year | year + sex + c | 64 | 110853.81 | 98.34 | 0.00 | 110725.24 |
| season × year + sex | season | . | . | year + sex | 62 | 110856.69 | 101.23 | 0.00 | 110732.16 |
| season × year + sex | season | . | . | year + sex + c | 63 | 110857.79 | 102.32 | 0.00 | 110731.24 |
| season + year + sex | season | . | . | year + sex | 54 | 110861.83 | 106.36 | 0.00 | 110753.42 |
| season + year + sex | season | . | . | year + sex + c | 55 | 110862.88 | 107.41 | 0.00 | 110752.46 |
| season × year + sex | season | year | . | year + sex | 71 | 110867.03 | 111.56 | 0.00 | 110724.33 |
| season × year + sex | season | year | . | year + sex + c | 72 | 110868.29 | 112.82 | 0.00 | 110723.57 |
| season × year + sex | season + year | year | year | year + sex | 88 | 110908.92 | 153.46 | 0.00 | 110731.85 |

^a^ We modeled the probability of recapture within a primary occasion (‘*c*’) given an initial capture as a function of the probability of initial capture (‘*p*’). In addition, we held the dead reporting rate (‘*r*’) constant and fixed at 0 (there were virtually no dead reports during our study, and the model structure of both the availability parameters (*a” and a’*) did not vary in this stage (month + year + sex).

^b^ Number of estimable parameters in the model.

^c^ Akaike’s information criterion corrected for small sample bias.

^d^ Akaike’s weight of evidence.

^e^ Variables in the model include: ‘season’ – division between seasons, where breeding includes April 15 to July 15, and non-breeding which includes July 15 to the following breeding season, ‘year’ – estimates vary among years of the study (2005–2014), ‘female’ – estimates vary by sex, ‘month’ – estimates vary across months (April–August), ‘.’ – constant, and ‘c’ – estimates of recapture rate are a function of initial capture rates.

^f^ Top-ranked model (lowest AIC_c_ value).

*Stage 2:* In the second stage of analysis, we included the effect of age (minimum known age from 1–10 yo) and reproductive output from the previous year on survival (*S*). We tested these structures for *S*, retaining the structure for the remaining variables (*a’, a”*, *F*, *p*, *c*, *R*, *R’, r*) from the top-ranked (lowest AIC_c_) model from stage 1.

| S | K ^a^ | AIC_c_ ^b^ | ΔAIC_c_ | Weight ^c^ | Deviance |
| --- | --- | --- | --- | --- | --- |
| season × year + sex × age ^d^ | 82 | 110861.17 | 0.00 | 0.58 | 110696.24 |
| season × year + sex + age | 81 | 110861.80 | 0.63 | 0.42 | 110698.89 |
| season + year + sex × age + Ro | 75 | 110875.71 | 14.54 | 0.00 | 110724.93 |
| season + year + sex + al + Ro | 74 | 110876.72 | 15.55 | 0.00 | 110727.96 |

^a^ Number of estimable parameters in the model.

^b^ Akaike’s information criterion corrected for small sample bias.

^c^ Akaike’s weight of evidence.

^d^ Variables in the model include: ‘season’ – division between seasons, where breeding includes April 15 to July 15, and non-breeding which includes July 15 to the following breeding season, ‘year’ – estimates vary among years of the study (2005–2014), ‘sex’ – estimates vary by sex, ‘age’ – estimates vary by bird age, measured as minimum known age in years (i.e., years since banding, or in the case of a bird banded at hatch, years since first appearance as an adult), and ‘Ro’ – estimates are related to the population average reproductive output the previous year.

*Stage 3:* In the third stage, we compared model structures for *a”* (probability of remaining available, or within season fidelity) that included variables for average nest failure rate during the 30-day period, age, and a linear trend over month. We tested these structures for *a”*, retaining the structure for the remaining variables (*S, a’,* *F*, *p*, *c*, *R*, *R’, r*) from the top-ranked (lowest AIC_c_) model from stage 2.

| a" | K ^a^ | AIC_c_ ^b^ | ΔAIC_c_ | Weight ^c^ | Deviance |
| --- | --- | --- | --- | --- | --- |
| year + Month + sex + nest ^d^ | 82 | 110842.83 | 0.00 | 0.54 | 110677.90 |
| year + month + sex + nest | 83 | 110844.81 | 1.98 | 0.20 | 110677.86 |
| year + Month + sex + nest + age | 83 | 110844.88 | 2.05 | 0.19 | 110677.93 |
| year + month + sex + nest + age | 84 | 110846.83 | 4.00 | 0.07 | 110677.85 |
| year + month + sex | 82 | 110861.22 | 18.39 | 0.00 | 110696.29 |
| year + month + sex + age | 83 | 110863.18 | 20.35 | 0.00 | 110696.23 |
| year + Month + sex + age | 82 | 110887.79 | 44.96 | 0.00 | 110722.86 |

^a^ Number of estimable parameters in the model.

^b^ Akaike’s information criterion corrected for small sample bias.

^c^ Akaike’s weight of evidence.

^d^ Variables in the model include: ‘year’ – estimates vary among years of the study (2005–2014), ‘month’ – estimates vary independently across months (April–August), ‘Month’ – estimates vary linearly across months (April–August) ‘sex’ – estimates vary by sex, ‘nest’ – estimates are related to the average nest failure rate in the population during the previous month, and ‘age’ – estimates vary by bird age, measured as minimum known age in years (i.e., years since banding, or in the case of a bird banded at hatch, years since first appearance as an adult).

*Stage 4:* In the fourth stage, we compared model structures for *a’* (returning from the unobservable state, or re-immigrating) that contained variables for reproductive output in the previous year, age, and a linear trend in month. We tested these structures for *a’*, retaining the structure for the remaining variables (*S, a”,* *F*, *p*, *c*, *R*, *R’, r*) from the top-ranked (lowest AIC_c_) model from stage 3.

| a' | K ^a^ | AIC_c_ ^b^ | ΔAIC_c_ | Weight ^c^ | Deviance |
| --- | --- | --- | --- | --- | --- |
| year + month + sex + Ro + age ^d^ | 84 | 110837.93 | 0.00 | 0.62 | 110668.95 |
| year + month + sex + age | 83 | 110840.14 | 2.22 | 0.20 | 110673.19 |
| year + month + sex + Ro | 83 | 110840.47 | 2.55 | 0.17 | 110673.52 |
| year + month + sex | 82 | 110853.57 | 15.64 | 0.00 | 110688.64 |
| year + Month + sex + age | 81 | 110857.58 | 19.65 | 0.00 | 110694.67 |
| year + Month + sex + Ro + age | 82 | 110858.39 | 20.46 | 0.00 | 110693.46 |
| year + Month + sex + Ro | 81 | 110859.14 | 21.21 | 0.00 | 110696.23 |

^a^ Number of estimable parameters in the model.

^b^ Akaike’s information criterion corrected for small sample bias.

^c^ Akaike’s weight of evidence.

^d^ Variables in the model include: ‘year’ – estimates vary among years of the study (2005–2014), ‘month’ – estimates vary independently across months (April–August), ‘Month’ – estimates vary linearly across months (April–August) ‘sex’ – estimates vary by sex, ‘Ro’ – estimates are related to the population average reproductive output the previous year, and ‘age’ – estimates vary by bird age, measured as minimum known age in years (i.e., years since banding, or in the case of a bird banded at hatch, years since first appearance as an adult).

*Stage 5:* In the fifth and final stage, we compared the top-ranked model from stage 4 to models with full time (monthly and yearly) variability in *a”* and *a’* (year × month + sex) using the analysis of deviance (Skalski 1996). We compared the fully time variable model to the baseline model (year + month + sex) and the baseline model with added time-specific covariates to determine the proportion of temporal variation described by the covariate model. All other variables were given the structure of the top-ranked model from Stage 4. Thus the ‘Covariate’ model is the top-ranked model from Stage 4 in both cases.

| Parameter | Model | Variables ^a^ | Deviance | Variation Described ^b^ |
| --- | --- | --- | --- | --- |
| a” | Baseline | year + month +sex | 110687.6 |  |
|  | Covariate | year + Month + sex + nest | 110668.9 | 11.8% |
|  | Time | year × month + sex | 110529.4 |  |
| a’ | Baseline | year + month +sex | 110688.6 |  |
|  | Covariate | year + month +sex + Ro +age | 110668.9 | 55.7% |
|  | Time | year × month + sex | 110653.2 |  |

^a^ Variables in the model include: ‘year’ – estimates vary among years of the study (2005–2014), ‘month’ – estimates vary independently across months (April–August), ‘Month’ – estimates vary linearly across months (April–August) ‘sex’ – estimates vary by sex, ‘Ro’ – estimates are related to the population average reproductive output the previous year, and ‘age’ – estimates vary by bird age, measured as minimum known age in years (i.e., years since banding, or in the case of a bird banded at hatch, years since first appearance as an adult)

^b^ The percent of temporal variation described by a time-dependent covariate. Defined as: $\frac{(\mathrm{Deviance}_{\mathrm{null}}-\mathrm{Deviance}_{\mathrm{covariate}})}{(\mathrm{Deviance}_{\mathrm{null}}-\mathrm{Deviance}_{\mathrm{Time}})}$ (Skalski 1996)
