# Supplementary material for: Senescence and carryover effects of reproductive performance influence migration, condition, and breeding propensity in a small shorebird
Source: Ecol Evol. 2017 Nov 15;7(24):11044–56. doi: 10.1002/ece3.3533 (PMC5743479; doi:10.1002/ece3.3533)
Supplement: Supplementary file 2 [file ECE3-7-11044-s002.docx]

Supplemental Information. Weithman et al. Senescence and Carryover Effects of Reproductive Performance Influence Migration, Condition, and Breeding Propensity in a Small Shorebird

**Appendix S2: Supplemental figures for manuscript**

Figure S1: Average seasonal survival of piping plovers on the Missouri River during the breeding (circles; approximately April 15–Jul 15) and non-breeding (triangles; approximately Jul 15–Apr 15) seasons (2005–2014). Error bars represent 1 SE.

Figure S2: Annual true survival of female (circles) and male (triangles) piping plovers on the Missouri River (2005–2014). Error bars represent 1 SE.
